# Shock Measurements Based on Pendulum Excitation and Laser Doppler Velocimetry: Primary Calibration by SI-Traceable Distance Measurements

**DOI:** 10.6028/jres.125.011

**Published:** 2020-04-10

**Authors:** Muhammad Y. Afridi, Jon Geist, Michael Gaitan

**Affiliations:** 1 Potomac Networks, 1301 Delaware Ave. SW # N716, Washington DC 20024, USA; 2 National Institute of Standards and Technology, Gaithersburg, MD 20899, USA

**Keywords:** accelerometer calibration, laser Doppler velocimeter, measurement uncertainty, pendulum, primary calibration, shock excitation

## Abstract

A new method is described to provide a primary calibration of shock measurements produced by a shock measurement system consisting of pendulum excitation and laser Doppler velocimetry. The method uses the laser Doppler velocimeter to determine the total distance traveled by a rigid block that slides along a Teflon (fluorocarbon) channel after being struck by a pendulum head, and the resulting distance is compared to the distance measured by an SI-traceable length measurement. The instantaneous velocity of the block is measured by the velocimeter and is used to calculate the displacement of the block by integrating the velocity data. The result is compared to the displacement measured using calibrated rulers and calipers. The method was applied to an independently calibrated commercial velocimeter for impact accelerations ranging from 2000 to 30,000 m/s2. The results of the independent mechanical displacement measurements agreed with those from the commercial velocimeter to within ±0.3 %, with better agreement above accelerations of order 10,000 m/s2 to within ±0.1 %. A conservative, upper-bound, uncertainty analysis included the effects of noise and other random errors, as well as type B estimates for systematic errors from occasional momentary demodulation failures (dropouts), use of a different number of rulers before and after shock distance measurement, and the relative frequency response of the velocimeter

## 1 Introduction

A transient mechanical (physical) excitation, for example, a pendulum strike, can be used to characterize velocimeters and accelerometers [1, 2], as well as dynamic force sensors [3]. Such a transient excitation is also known as a mechanical shock, which creates a sudden acceleration and significant relative displacement of physical objects that are free to move on their supporting surfaces. The shock itself can be characterized in terms of its peak acceleration, shape, and duration [4, 5], whether or not the object is free to move.

Accelerometers are used in a wide range of applications, including inertial navigation systems, cell phones, cameras, drones, automobiles, wearable devices, and video game controllers, and they are increasingly used in devices based on microelectromechanical systems (MEMS) technologies. There is also a trend toward developing acceleration sensors with higher performance, driving a need to improve on the accuracy and range of traceable vibration and shock measurements.

In 1999, Martens *et al*. [6] reviewed the current state-of-the-art and trends for ensuring traceability for vibration and shock measurements with expanded uncertainties (*k* = 2) of measurement for shock-shaped acceleration of 0.3% to 1% for peak accelerations ranging from 1000 m/s^2^ to 50,000 m/s^2^ using laser interferometry. Link *et al*. [7] presented a signal processing method for determining the dynamic behavior of accelerometers by shock excitation and laser interferometry. Their signal processing technique was adopted by the International Organization for Standardization (ISO) Technical Committee (TC) 108/SC 3 as a primary shock calibration method specified in ISO 16063-13 *Primary Shock Calibration Using Laser Interferometry* [8]. To establish traceability in vibration and shock, the three translational motion quantities, *i.e.*, acceleration, velocity, and displacement, and the three rotational motion quantities, *i.e.*, angular acceleration, angular velocity, and rotation angle, must be realized (generated and measured by a primary measurement method) [6].

For primary shock measurements that are traceable to International System of Units (SI) units, the traceability path will often include a laser interferometer characterized by a scale factor that is traceable to the SI through laser wavelength and electrical frequency standards. One popular type of laser interferometer is the laser Doppler velocimeter (LDV).^1^1 The types of laser Doppler velocimeters that are used for acceleration measurements are sometimes called laser surface velocimeters or laser heterodyne velocimeters.

The calibration method for an LDV specified in ISO 16063-41[9] requires a comparison measurement between it and a primary laser homodyne interferometer, where both are directed onto the surface of a vibration exciter. The vibration exciter is swept over a range of frequencies, 0.4 Hz to 50 kHz or wider, and the results are compared using a sine approximation to determine the calibration factor for the LDV. This makes the calibration of the LDV a secondary calibration; however, in practice, the uncertainties that arise from calibration of an accelerometer under sinusoidal excitation are dominated by other factors compared to the uncertainties attributed to the LDV.

Under optimum conditions, such as digital demodulation and near-ideal periodic motion with its built-in redundancy, the LDV approach is so accurate that comparison with, or calibration by, other approaches has not been considered either necessary or practical. However, with less ideal excitation lacking redundancy, such as a shock impulse, errors in laser wavelength and beat frequency are not necessarily the limiting sources of uncertainty in the measurement. Instead, the performance of the demodulator, which is much more difficult to characterize, can limit the uncertainty.

Here, we demonstrate that for a reliable uncertainty statement for the results of shock measurements, it is both necessary and practical under certain circumstances to calibrate the results of each measurement. The LDV that we used was carefully calibrated by the manufacturer and uses a very well-tested digital demodulation subsystem optimized for measuring velocities of scattering surfaces over large distances. The uncertainty stated by the manufacturer is ±1% for signal near full scale to allow for the possibility of larger errors in real measurement environments.

However, some of the LDV output signals we measured suffered from a type of demodulation error called dropout error. We wrote a program to correct these errors but needed an independent way to calibrate the scale factor of the LDV, including uncertainty estimates both before and after correction.

In this work, we present a method for directly calibrating the results of pendulum-based, LDV shock measurements by comparing the mechanically measured displacement (distance) of a test block that moves in response to a shock excitation with the displacement obtained by integrating the instantaneous velocity of the test block as measured by the LDV. This report focuses on LDVs, but the method can be applied to other types of interferometers as well.

For peak accelerations from approximately 1500 to 32,000 m/s^2^, agreement was well within the ±0.5% maximum linearity error specified by the manufacturer for the velocimeter used. After correction for dropouts, which cannot be corrected by integration or a linear filter because their effect is not symmetric around the true signal, the displacements calculated by integrating the instantaneous velocities measured by the LDV agreed with the displacements determined mechanically to within ±0.1% above 10,000 m/s^2^.

Although these results might be expected due to the good agreement obtained among laboratories participating in international comparisons of shock and acceleration measurement capability, this kinematic approach provides a completely independent path to link the measured shock time series to SI in a suitably equipped shock calibration laboratory.

Finally, the method is particularly convenient for measurements of accelerometers with digital outputs because the method calibrates the entire measurement system, leaving only the relative frequency response of the LDV to be determined. Our results show that the zero frequency (DC) response is accurate to within ± 0.1% and that the frequency content of our signals was negligible above 50 kHz. The specified frequency response of the LDV that we used was greater than 0.94 at 1.5 MHz. This corresponds to a gain error of 2 parts in 10^6^ at 50 kHz, which is negligible at the 0.1% level.

## 2 Kinematics

The instantaneous acceleration *a(t)* measured by an ideal linear accelerometer when moved a displacement *D(t₁,t₂)* along its axis of maximum sensitivity during a time interval *t₁ ≤ t ≤ t₂*

$a(t)=K\left[Z_{a}\left(t\right)-B\right]$,(1)

Where *Zₐ(t)* is the output signal ^2^2The output described can be expressed in whatever units an instrument provides without any corrections or unit conversions. from the accelerometer, *B* is a constant background signal (offset) from the accelerometer, and *K* is the accelerometer calibration factor. The calibration factor of an ideal linear accelerometer can be determined as

$K=\frac{D\left(t_{1},\;t_{2}\right)}{\int_{t_{1}}^{t_{2}}\left[\int_{t_{1}}^{\tau }\left[Z_{a}\left(t\right)-B\right]dt\right]d\tau }$ ^,^ (2)

where τ is a dummy variable of integration in the second integral with respect to the time of the second factor in Eq. (1), the distance $D\left(t_{1},\;t_{2}\right)\;$is measured with rulers and calipers traceable to the SI definition of the meter, and the time t is traceable to the SI definition of the second through the time base of a high-quality data acquisition system.

While conceptually simple, this displacement-based calibration method is seldom, if ever, implemented for shock, apparently because accelerometers suitable for shock measurements deviate substantially from ideal conditions due to instabilities in the background *B*. Such instabilities are magnified by the first integration and then further magnified by the second integration and result in large uncertainties. Other calibration methods such as LDVs can provide much smaller uncertainties.

Modern LDVs can accurately measure shock-induced velocity of a light-scattering surface as a frequency-modulated signal given by

$v\left(t\right)=\frac{\lambda \;f(t)}{2}$, (3)

where λ is the laser wavelength, f is the modulated signal, and v is the projection of the instantaneous velocity of the surface onto the LDV laser beam. Research instruments that measure the modulation frequency with spectrum analyzers have been described at least as early as 1963 [10], but their actual implementation and operation differ markedly from the commercial LDVs that have been available for fluid flow since 1981 and for vibrometry and other applications since 1990. LDVs based entirely on fiber-optics technology, generally called photonic Doppler velocimeters (PDVs), are also widely used, with uncertainties of the order of ±1% being readily achievable [11]. While modern LDVs are capable of very high accuracy, they are not as easy to calibrate as Eq. (3) might suggest [6]. This is particularly true if the LDV output being used is either temporally complex or analog, whether from an analog or digital demodulator (velocity decoder).

If the optical axis of an LDV is aligned with the axis of motion of the velocimeter, then the calibration constant in Eq. (1) is given by

$K_{LDV}=\frac{D(t_{1},t_{2})}{\int_{t_{1}}^{t_{2}}\left[Z_{v}\left(t\right)-b\right]dt}$

where $Z_{v}\left(t\right)$ is the raw output velocity signal of the LDV, and *b* is the nominally constant background signal (DC offset) of the LDV. If an LDV-based shock measurement system is configured as described in the next section, and if the total distance travelled$\;D(t_{1},t_{2})$ can be accurately measured, then it is convenient to calculate the acceleration signal $Z_{a}\left(t\right)$ in Eq. (1) as the derivative with respect to time of $Z_{v}\left(t\right)$ and to determine *K*= $K_{LDV}$ in Eq. (1) from Eq. (4).

One advantage of this approach is that it combines the short traceability chain for acceleration of Eq. (1) with the near-ideal linearity and offset of the LDV. A second advantage is that only one integration of the signal is required for the calibration, and only one differentiation is required to calculate the desired acceleration signal, rather than two differentiations from a direct LDV measurement of distance. This is important because the biggest source of error in acceleration data calculated from distance data is the multiplication of small frequency components of noise by (2 πf) ^2^. Similarly, the biggest source of error in distance data calculated from measured acceleration data is the twice-integrated DC-offset signal *B*. A single integration and a single differentiation were determined to be more reliable than either of the other calibration/measurement options.

## 3 Shock Measurement System

**Fig. 1:**
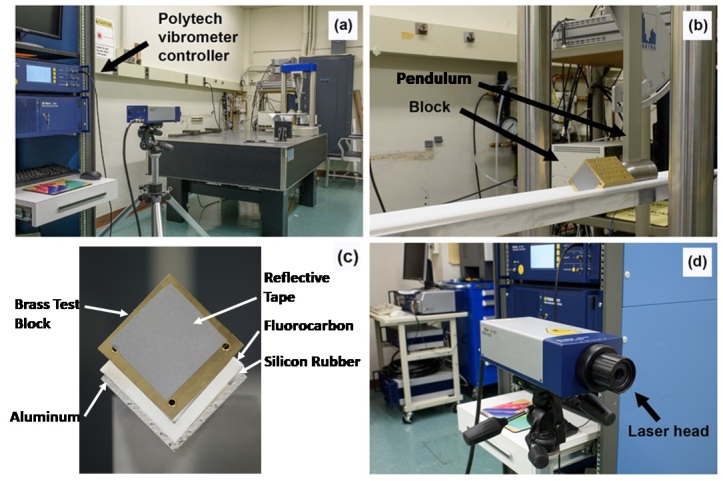
The primary shock calibration system, including (a) the Polytec vibrometer controller, (b) the brass block with reflective tape at rest on the V-shape rail with Teflon surface material, (c) the V-shape railing with the solid brass test block (front view), and (d) the Polytec Laser sensor head.

Figure 1 shows the calibration setup that we developed and used to demonstrate the practicality and low uncertainties available from primary velocimeter calibrations of pendulum shock testers by mechanical-contact measurements of displacement. The goal was not to optimize the system to minimize the uncertainty but to establish an upper level of uncertainty that can be improved by optimization for specific applications.

The key hardware components ^3^3 Certain commercial equipment, instruments, or materials are identified in this paper to foster understanding. Such identification does not imply recommendation or endorsement by the National Institute of Standards and Technology (NIST), nor does it imply that the materials or equipment identified are necessarily the best available for the purpose
in this setup are a Polytec OFV-5000 vibrometer controller, a Polytec OFV-503 laser sensor head, a NIST-calibrated, 24 bit data acquisition system (DAS) from National Instruments (NI) PXI 5922, a Spektra pendulum shock exciter, a 1.2 m long fluorocarbon (Teflon) V channel fastened with silicon-rubber adhesive onto a supporting aluminum frame, and a solid brass test block of dimensions 5.08 cm (W) × 5.08 cm (H) × 7.62 cm (L).

The test block is positioned in the V-channel and is free to slide with a maximum displacement of 850 mm when struck by the pendulum head. The fluorocarbon has a static coefficient of friction that is approximately equal to the kinetic coefficient of friction, which is approximately independent of velocity. However, the V-channel turned out to be not precisely square, so the test block rode on one edge and on one side of the V-channel, as shown in Fig. 1(c).

A commercial piece of gray light-scattering tape is attached to the side of the block facing the LDV. One or more pieces of soft tape are attached to the other side of the block facing the pendulum head to vary the duration of the shock pulse. The reflective tape produces a velocity signal that is less prone to dropout errors, described in the next section, when compared to the bare test block. The soft tape not only controls the shape of the pulse, but it also damps ringing generated by impact with the pendulum head. A thin metal strip is taped on one side at the bottom of the V-channel to set the starting position of the block, such that the pendulum head touches the soft tape when the test block is in contact with the metal strip.

The vibrometer controller is configured with a Polytec VD-09 decoder (demodulator), which can measure a maximum velocity of ±10 m/s. The controller is set to the 1 m/s/V velocity range with maximum frequency of 1.5 MHz, and all optional electronic filters are bypassed. The unit is configured to output velocity data as a voltage on its analog output port. The laser sensor head is mounted on a tripod, and the laser beam is aligned collinear with the axis of the V-channel near the center of the reflective tape on the test block. The tripod is placed approximately 1.5 m from the end of the V-channel, and the laser beam is manually focused for maximum depth of field at this distance.

The DAS (NI PXI 5922) was configured for direct coupled input and a 5 V range with a sampling rate set for 1 megasamples per second (1 MS/s) with 22 bit resolution. The DAS was programmed to capture the transient signal during a period of 3 s.

## 4 Procedure

To start a calibration, the test block is placed against the metal strip that is attached to the V-channel. The distance from the end of the V-channel (which will be shortened to “location”) is measured three times as follows. Two calibrated 304.80 mm rulers and one calibrated 152.40 mm ruler are laid end-to-end with one end in contact with the test block. The remaining 97 mm section to the end of the V-channel is measured independently three times with the probe of a calibrated 152.4 mm digital caliper having a resolution of 0.01 mm. A correction for nonideal contact between the rulers is applied when required as discussed in the next section.

To capture the velocity curves, the pendulum is lifted by hand and pressed against a stop set at a desired starting angle, the data acquisition is started, and the pendulum is released to strike the soft tape in the center of the test block. The test block slides along the V-channel and comes to a stop at some point along the V-channel. The instantaneous velocity of the test block is captured by the velocimeter, and its analog output velocity signal is digitized by the NI data acquisition system at 1 MS/s under the control of a MATLAB program. The resulting velocity curve is stored on a computer hard disk for data analysis.

The same procedure that was used to measure the block’s starting position is used to measure its position after it comes to a stop. However, depending upon how far the test block moved, only one or two of the rulers may be needed to position the ruler within the reach of the 150 mm caliper. The displacement of the test block is calculated as the location of the test block that was measured before the shock event minus the location of the test block that was measured after the shock event. If the same rulers are used for the before- and after-shock measurements, then the systematic errors associated with initial and final measurements cancel, and the net effect of the contact is to cause variations in the before-shock and after-shock location measurements.

**Fig. 2:**
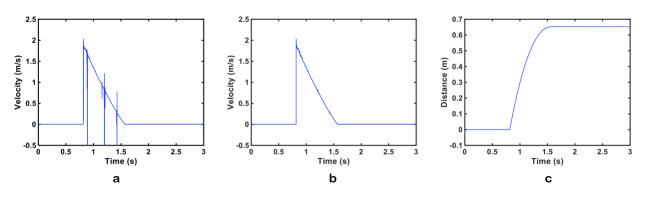
(a) The velocity data measured by the velocimeter following removal of the DC offset. This data set contains dropout errors. (b) The velocity data v(t) following dropout correction. (c) The distance as a function of time obtained by integrating v(t).

Figure 2(a) shows a typical raw velocity curve obtained from the velocimeter. We note that the narrow spikes in the velocity are not real changes in velocity. Instead, they are decoder dropout errors, which are caused by changes in the laser-speckle pattern when the solid test block is in motion. When a sufficiently dark portion of the speckle pattern passes over the velocimeter photodiode, the demodulator loses the modulated signal, and an erroneous signal is generated, which is characterized by the term “dropout.” As a matter of principle, the dropouts should not be filtered by a linear filter because this will introduce error in the integral of the signal, since the dropout signal is not symmetric about the true instantaneous velocity signal.

A MATLAB program was written to correct the dropout errors by replacing them with an average value calculated from the dropout-free regions adjacent to each of the dropouts. Figure 2(b) plots the velocity of the test block calculated by applying this dropout correction to the raw velocity data in Fig. 2(a).

Before dropout correction, the small, but nonzero DC offset of the velocity data was replaced by its average value obtained before the impact of the pendulum.

To calibrate the velocimeter with the mechanical-contact measurements described above, it is necessary to integrate the velocity data to distance as a function of time and determine the total displacement by subtracting the value after the test block has stopped moving from the value prior to the pendulum striking the test block. For integration, a MATLAB function (CUMTRAPZ) based on cumulative trapezoidal numerical integration was used. Prior to integration, any residual DC offset present in the velocity curve was removed by subtracting the mean value calculated from the velocity data before the impact of the pendulum. Figure 2(c) shows the distance curve as a function of time for the dropout-corrected velocity curve given in Fig. 2(b).

## 5 Velocity Time-Series Calibration by Distance Measurements

In total, 24 independent shock experiments were carried out. Each experiment consisted of three parts: (1) Prior to the shock event, three independent mechanical-contact measurements of the distance from the end of the V-channel to the test block (location of the test block) were made using calibrated machinist rulers and calipers; (2) during the shock event, a measurement of the analog signal from the velocimeter yielding the instantaneous velocity $v\left(t\right)$ was sampled at 1 MS/s to fully capture the shock event from start to finish; and (3) after the shock event, three independent mechanical-contact measurements of the location of the test block were made.

For each of the 24 independent experiments, the difference $\Delta_{vm}$ between the baseline-corrected displacement calculated from the velocimeter data, $s_{v}$, and the displacement measured by mechanical contact, $s_{m}$, was calculated as

${\;\Delta }_{vm}=s_{v}-s_{m}$, (5)

where $s_{v}=\int_{t=t_{0}}^{t_{1}}v(t)\;dt$, (6)

and $s_{m}=\frac{\left(s_{4}+s_{5}+s_{6}\right)}{3}-\frac{\left(s_{1}+s_{2}+s_{3}\right)}{3}$, (7)

where $t_{0}=0,\;t_{1}$ is chosen to be a sufficient amount of time for the event to be captured, $s_{1}$, $s_{2},\;$and $s_{3}$ are the three measurements of the test-block location before the shock event, and $s_{4}$, $s_{5}$, and $s_{6}$ are the three measurements of the test-block location after the shock event. In this work, $t_{1}$was set to 3 s.

As mentioned previously, the purpose of this report is to demonstrate that the scale factor of laser velocimeter measurements relative to SI units can be determined with a state-of-the art uncertainty [12, 13] by mechanical-contact measurements in a pendulum shock-measurement system such as ours. Therefore, only the uncertainty in $s_{m}$ is relevant, because we are determining a calibration factor for $s_{v}$ that is equivalent to the calibration factor for the velocity v(t) measured by the velocimeter.

For each shock event, the standard uncertainty $u_{m}$ (coverage factor *k* = 2) in $s_{m}$ was estimated as

$u_{m}=\sqrt{{u^{2}}_{A}+{u^{2}}_{B}}$, (8)

where $u_{A}$ is a type A uncertainty that describes the reproducibility of the location measurements, and $u_{B}$ is a type B uncertainty dominated by a correction for nonideal contact of the measurement instruments used for the location measurements.

To estimate $u_{B}$, we calibrated the calipers and rulers that were used in the experiments. The calibrations were carried out following standard procedures with a set of gauge-blocks traceable through NIST standards to the SI definition of the meter. The jaws of the calipers were pushed tightly against the gauge blocks and the rulers. The nominal lengths of the three rulers and the nominal readings of the two calipers were found to require no correction with an uncertainty well under 0.0002 mm, which is negligible compared to the type A uncertainties discussed next.

The measurements of the starting location of the test block for the 24 independent experiments were used to estimate $u_{A}$. The standard deviation of these measurements was $\sigma_{R}$ = 0.033 mm. Using this as input, the type A component of the uncertainty (*k* = 2) was estimated as

$u_{A}=2\sqrt{\frac{{\sigma^{2}}_{4}+{\sigma^{2}}_{5}+{\sigma^{2}}_{6}}{3}+\frac{{\sigma^{2}}_{1}+{\sigma^{2}}_{2}+{\sigma^{2}}_{3}}{3}}=2\sqrt{\frac{{{6\;\sigma }^{2}}_{R}}{3}}=2\sqrt{{{2\sigma }^{2}}_{R}}$, (9)

where it was assumed that $\sigma_{1}=\sigma_{2}={\ldots =\sigma }_{6}=\sigma_{R}$.

For the distance measurements, the rulers and a caliper were positioned by eye until they just contacted the test block or each other with a contact pressure that did not displace the test block. It was not possible to contact them more firmly against the test block without moving it, nor was it possible to press the caliper probe more tightly against the ruler during these measurements.

A correction for loose stacking of rulers in the measurement of $\Delta_{vm}$ was approximated as

$\Delta_{T}=\Delta_{L}\left(\delta_{4j}+\delta_{5j}+\delta_{6j}-\delta_{1j}-\delta_{2j}-\delta_{3j}\right)$, (10)

where ${∆}_{L}\;$was estimated to be 0.01 mm with a type B standard uncertainty based on multiple measurements of a 3.24 cm gauge block with the calibrated calipers, with loose contact followed by firm contact.

The type B standard uncertainty (*k* = 2) for this error was given by

${u_{B}=2\sigma }_{T}=\left|\Delta_{T}\right|$, (11)

where $\delta_{ij}$ was equal to 1.0 if ruler j was used in the measurement of location $s_{i}$, and 0 if that ruler was not used. It is important to note that if the same rulers were used to measure both the initial and final locations of the text block, both the correction for loose stacking and its uncertainty would be zero because their effect is cancelled, and only the type A uncertainty associated with the variability of the contact remained. On the other hand, if different rulers were used, the correction was applied, and the uncertainty increased accordingly.

Note that $u_{B}$ does not describe the effect of lack of reproducibility of the length of the rulers when stacked together, which is included in $u_{A}$; it only describes our inability to precisely estimate the magnitude of the systematic error in the length of two rulers when not tightly stacked.

**Fig. 3:**
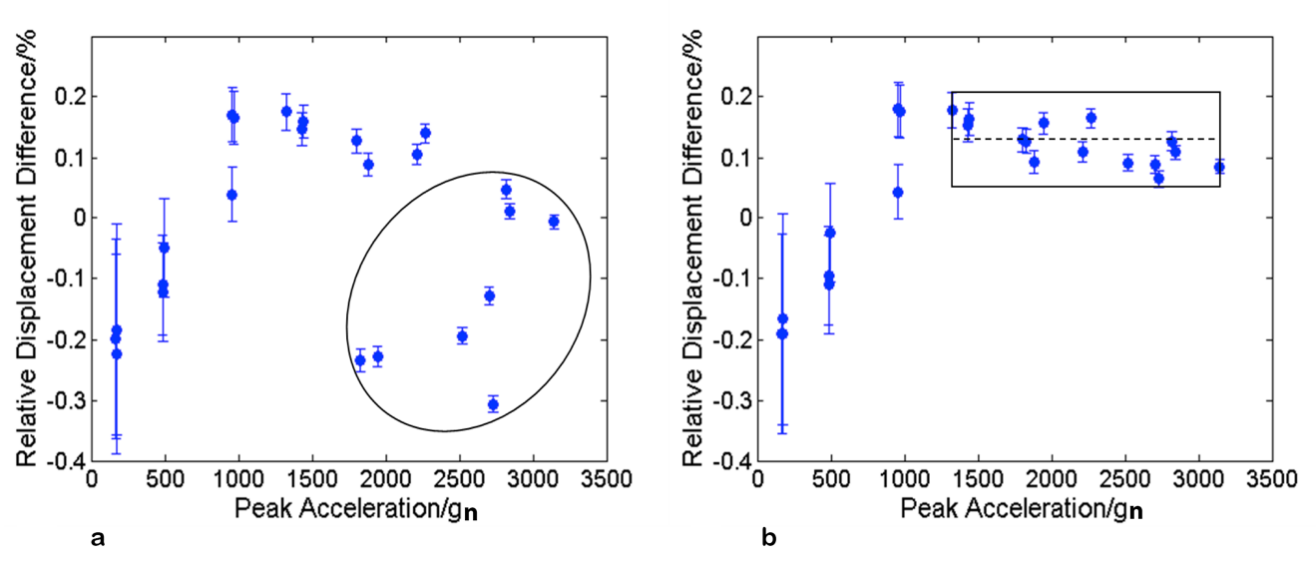
The relative differences between the 24 measurements of the displacement of a test block following a pendulum strike as measured with an LDV and with calibrated rulers and calipers. (a) No dropout correction was applied to the velocity data prior to integration to distance ($s_{v})$. (b) The dropout errors in the velocity data were removed prior to integration to distance ($s_{v})$. The rather serious looking dropout errors shown in Fig. 2(a) produce rather small and readily corrected errors in the distance calculated from the velocimeter output due to their low intrinsic width and the high sampling rate used. At accelerations below about 1300 *g_n_*, ^4^4 Here, gn refers to the standard acceleration due to gravity defined as 9.80665 m/s2, from The International System of Units (SI)—Conversion Factors for General Use, NIST Special Publication 1038, May 2006, which is included in our discussion because it is often used in the expression of acceleration measurements. the uncertainty is dominated by the relative uncertainty in the mechanical measurements of small displacements of the test block. The 10% point shock durations varied from approximately 500 μs at the lowest peak acceleration to 100 μs at the largest peak accelerations.

Equations (5) through (11) were applied to the data recorded for each shock measurement. Figure 3 plots the relative differences, $\Delta_{vm}/s_{m}$, between the displacements determined from the velocity measurements and those determined from the mechanical-contact measurements versus the peak acceleration of the shock, which was calculated as

$a=max\left(f_{50}\left[\frac{{dv}_{c}(t)}{dt}\right]\right)$, (12)

where the operator $f_{50}$ [..] applies a zero-phase-shift, 50 kHz cutoff, low-pass Butterworth filter to its argument. The filter details are given in the data processing and zero-phase, digital low-pass filtering section.

Figure 3(a) plots $\Delta_{vm}/s_{m}$ following the removal of DC offset from the raw velocity data, but without the correction for dropout error. Figure 3(b) plots $\Delta_{vm}/s_{m}$ following removal of DC offset and the dropout correction. The error bars in these figures are the standard uncertainty estimates $u_{m}$ (*k* = 2) that were described above.

A comparison of Fig. 3(a) and (b) shows that the effect of dropout errors on the relative differences enclosed in the ellipse in Fig. 3(a) is very large with respect to the estimated standard uncertainties $u_{m}$ in the mechanical-contact measurements above 1000 *g_n_*, even though all relative differences fall well within ±0.4%. However, after dropout correction, all the relative differences for peak accelerations in the range from approximately 1323 *g_n_* to 3144 *g_n_*, which are shown in the rectangle in Fig. 3(b), have a mean of 0.12% with a range less than ±0.1%.

A straight-line fit of the data in the rectangle gives

R(a)= 0.00121 ± 0.00013 – (4.3 ± 2.3)×10^-7^ (a – 2250 *g*_n_), (13)

which describes the slope of the data in the rectangular box in the rectangular box in Fig. 3(b), where R(a) is the relative difference, a is the peak acceleration, and the coverage factor of the standard errors is *k* = 2. The small slope is very likely an uncorrected systematic error associated with either the distance measurements or the velocimeter output. We conjecture that it is an error arising during the dropout correction.

Table 1 compares misleadingly conservative and more moderate calibration factors and related uncertainty estimates for the shock measurement system described here. These values are comparable to the best currently available from commercial instruments such as the one that we used.

**Table 1:** Calibration factor (reciprocal of ratio plotted in Fig. 3b) and measurement uncertainties for different range of velocities.

Calibration	Range (m/s^2^)	Calibration Factor	Uncertainty (*k* = 2)
Misleadingly Conservative	200–3150	0.999	±0.003
Moderate	200–1300	0.999	±0.003
Moderate	1000–3150	1.001	±0.001

## 6 Data Processing and Digital, Zero-Phase-Shift, Low-Pass Filtering

**Fig. 4:**
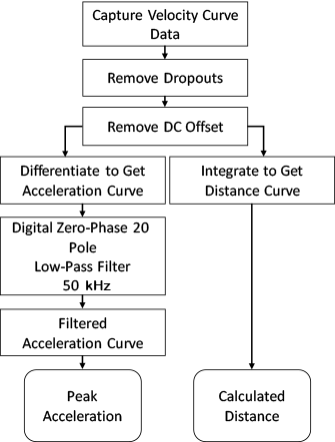
Block diagram of velocimeter data processing to get distance and peak acceleration values.

**Fig. 5:**
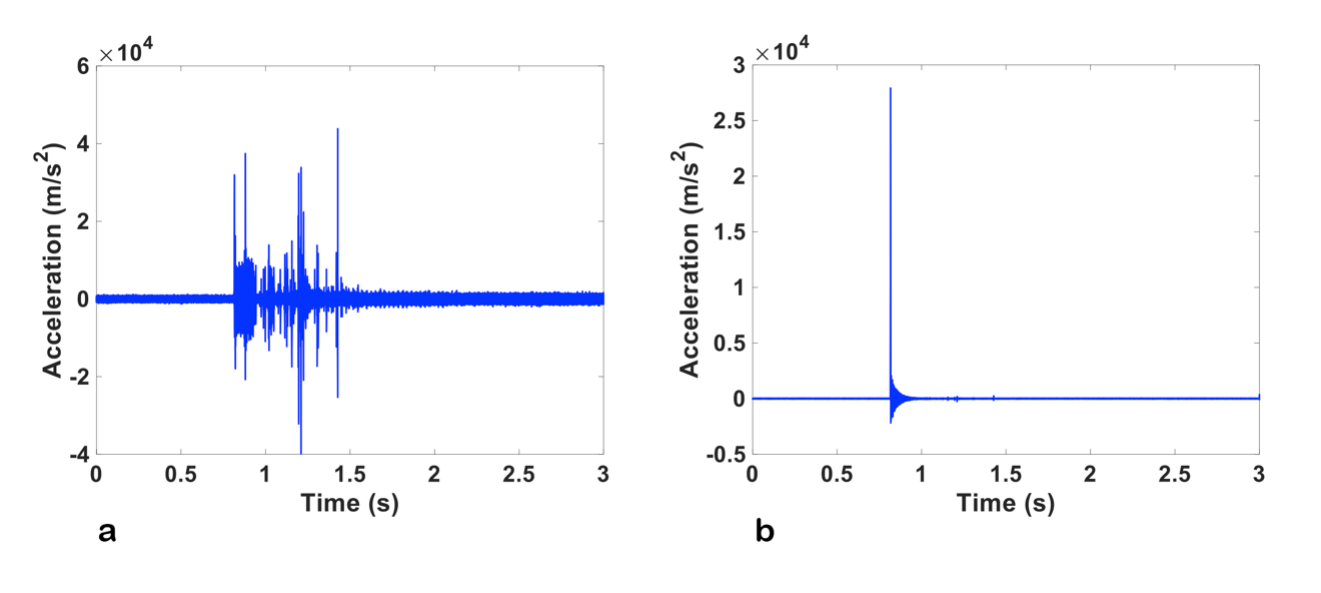
(a) Unfiltered acceleration obtained from the corrected velocity data and (b) acceleration curve after filtering with 50 kHz cutoff filter described above.

Figure 4 shows a block diagram of data processing to get distance and peak acceleration values from a velocimeter’s velocity curve recorded during a shock event. Figure 5(a) shows the acceleration data obtained by differentiating the corrected velocity data as mentioned previously. The acceleration data obtained in this way are noisy and hide the actual acceleration pulse. To remove the noise, two digital 20 pole Butterworth low-pass filters were designed and implemented in the MATLAB programming environment. One had with a 20 kHz cutoff frequency and the other had a 50 kHz cutoff frequency. The digital filter coefficients were used in the MATLAB filter function called FILTFILT. After filtering the data in the forward direction, the FILTFILT function reverses the filtered sequence and runs it back through the filter. The net result of using the FILTFILT function is that it doubles the order of the filter, in this case, to 40 poles, with zero-phase distortion and a flat amplitude curve from the reciprocal of the sampling time to within a few hertz of the cutoff frequency. The filtered data are shown in Fig. 5(b).

**Fig. 6:**
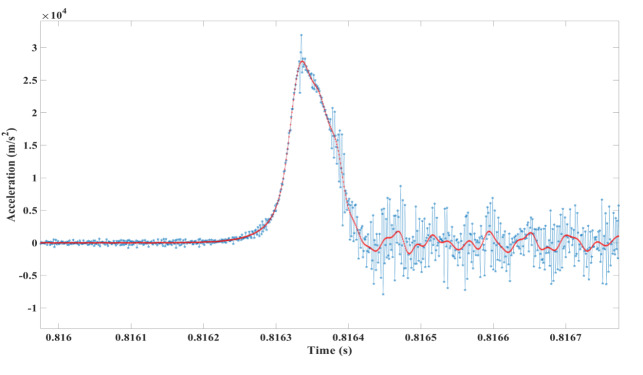
A comparison of the results of filtering the acceleration data in Fig. 5a with the 20 kHz and a 50 kHz filters described above.

Figure 6 compares close-up versions of the filtered acceleration data obtained by forward/backward filtering the acceleration data shown in Fig. 5a with 20 kHz cutoff and 50 kHz cutoff, zero-phase-shift, 20 pole, Butterworth, low-pass digital filters. The fact that the results from the 20 kHz filter accurately follow the results of the 50 kHz filter on the steep region of rising acceleration show that there are no frequency components between 20 kHz and 50 kHz in the unfiltered acceleration signal in Fig. 5a. This is an expected result of the use of damping tape on the struck side of the test block. Since the damping removed all frequencies between 20 kHz and 50 kHz it also removed all frequencies above 50kHz.

## 7 Conclusion

We have described a method for SI-traceable calibration of acceleration time series derived from LDV measurements of shock-induced, test-block motion, where the instantaneous displacement is determined by integrating the instantaneous velocity signal. The transient velocity signal is then integrated to calculate the total displacement, and the result is compared to SI-traceable distance measurements to determine the calibration factor of the LDV. This calibration method is well suited for shock measurement systems that include an LDV that produces a velocity time series for a test block on which an accelerometer under test is mounted and that moves along a linear track and comes to a stop due to frictional forces. The SI-traceable calibration factor is given as a ratio of the distance traveled by the block as measured by rulers and calipers divided by the first integral of the velocity signal from a convenient time just prior to the shock event to a convenient time following the event.

The major sources of error associated with determining the calibration factor include nonnegligible DC offset and velocity-curve dropout features, as well as noise and uncertainties in the mechanical measurement of the distance that the test block traveled. Examples of each and their correction, including an uncertainty analysis, were presented. The method was applied to an independently calibrated commercial velocimeter for impact accelerations ranging from 2000 to 30,000 m/s^2^. The results of the independent displacement measurements agreed within a range of ±0.3%. Above 10,000 m/s^2^, our measured calibration constant was 1.001 (m/s)/V within a range of ±0.1%.

Where applicable, this SI-traceable calibration method of an acceleration time series may prove to be less expensive to implement, more convenient to carry out, and more accurate than other accepted methods. It can also be used to provide an independent primary calibration of a velocimeter or an independent check of a primary calibration of a velocimeter. In either case, if a shock system that includes a moving test block is not readily available, then mechanical calibration with a suitable rotating system, such as that described in Refs. [14] and [15], may prove useful and simple to implement.
